# Evaluation of the Gamma-H2AX Assay for Radiation Biodosimetry in a Swine Model

**DOI:** 10.3390/ijms140714119

**Published:** 2013-07-08

**Authors:** Maria Moroni, Daisuke Maeda, Mark H. Whitnall, William M. Bonner, Christophe E. Redon

**Affiliations:** 1 Armed Forces Radiobiology Research Institute, Uniformed Services University, Bethesda, MD 20889-5603, USA; E-Mails: maria.moroni@usuhs.edu (M.M.); mark.whitnall@usuhs.edu (M.H.W.); 2 Laboratory of Molecular Pharmacology, Center for Cancer Research, National Cancer Institute, National Institutes of Health, Bethesda, MD 20892, USA; E-Mails: maedad@mail.nih.gov (D.M.); bonnerw@mail.nih.gov (W.M.B.)

**Keywords:** Gottingen minipig, immunocytofluorescence-based γ-H2AX, ionizing radiation, lymphocytes, fibroblasts

## Abstract

There is a paucity of large animal models to study both the extent and the health risk of ionizing radiation exposure in humans. One promising candidate for such a model is the minipig. Here, we evaluate the minipig for its potential in γ-H2AX-based biodosimetry after exposure to ionizing radiation using both Cs137 and Co60 sources. γ-H2AX foci were enumerated in blood lymphocytes and normal fibroblasts of human and porcine origin after *ex vivo* γ-ray irradiation. DNA double-strand break repair kinetics in minipig blood lymphocytes and fibroblasts, based on the γ-H2AX assay, were similar to those observed in their human counterparts. To substantiate the similarity observed between the human and minipig we show that minipig fibroblast radiosensitivity was similar to that observed with human fibroblasts. Finally, a strong γ-H2AX induction was observed in blood lymphocytes following minipig total body irradiation. Significant responses were detected 3 days after 1.8 Gy and 1 week after 3.8 and 5 Gy with residual γ-H2AX foci proportional to the initial radiation doses. These findings show that the Gottingen minipig provides a useful *in vivo* model for validation of γ-H2AX biodosimetry for dose assessment in humans.

## 1. Introduction

Humans are increasingly exposed to ionizing radiation (IR) through routine clinical therapeutic procedures and radiological accidents [1–3]. Acute exposure to high doses of radiation rapidly leads to major injuries to the immune system and gastrointestinal tract [4,5]. In addition, long-term effects of radiation exposure include major health issues ranging from fibrosis to cancer [1,6,7]. Appropriate clinical treatments such as general medical management (barrier nursing conditions, administration of antibiotic, antimycotic and antiviral substances), administration of cytokines for bone marrow stimulation (*i.e.*, G-CSF), growth factors, antioxidants as well as stem cell therapy [8], could mitigate some of these effects. However, treatment decisions require accurate estimations of radiation exposure which are currently unavailable due to the lack of appropriate biodosimetry tools. One step towards validating more biodosimetry tools is the development of more convenient animal models for radiation biodosimetry as well as studying the acute radiation syndrome [9–14]. Current models utilizing non-human primates have many disadvantages including high cost, reduced availability, and ethical concerns [15]. One large animal species becoming more popular for biomedical research is the pig [16,17], since it is similar to the human in various aspects of its anatomy, physiology, metabolism, histopathology and pharmacodynamics. Hundreds of breeds are available worldwide. One, the Gottingen minipig or miniature swine [18], is currently being developed as a model to study and treat the effect of ionizing radiation in humans [12,14,19]. Studies show that the acute radiation syndrome in the minipig presents characteristics similar to those observed in humans [14]. A review on the use of swine in radiological research can be found elsewhere [20].

One immediate effect of radiation exposure is the formation of DNA double-strand breaks (DSBs), considered to be one of the most damaging DNA lesions. High DSB levels can lead to cell death while low levels may induce cellular senescence or genomic rearrangements that lead to cancer [21]. DSBs can be identified and quantified *in situ* by detecting the γ-H2AX foci formed at DNA break sites utilizing immunostaining techniques [22,23]. Counting γ-H2AX foci is the most sensitive of current assays for irradiation-induced DSBs [21] with a ratio of DSBs to visible γ-H2AX foci close to 1:1 [24,25].

In this study, we evaluate the suitability of the minipig model for γ-H2AX-based radiation biodosimetry by first examining lymphocytes and fibroblasts from minipigs exposed *ex vivo* to ionizing radiation as well as lymphocytes from pigs exposed to total body irradiation from a Co60 source. While peripheral blood lymphocytes are by far the prime choice for γ-H2AX-based radiation dosimetry *in vivo*, cultured fibroblasts have been used extensively *in vitro* to determine radiation sensitivity [26–29]. The use of these cells allowed us to assess the relationship between radiation sensitivity and γ-H2AX kinetics in both human and minipig models.

## 2. Results and Discussion

### 2.1. Comparative Analysis of γ-H2AX Kinetics in Human and Minipig Lymphocytes after *ex Vivo* Irradiation

**Figure 1:**
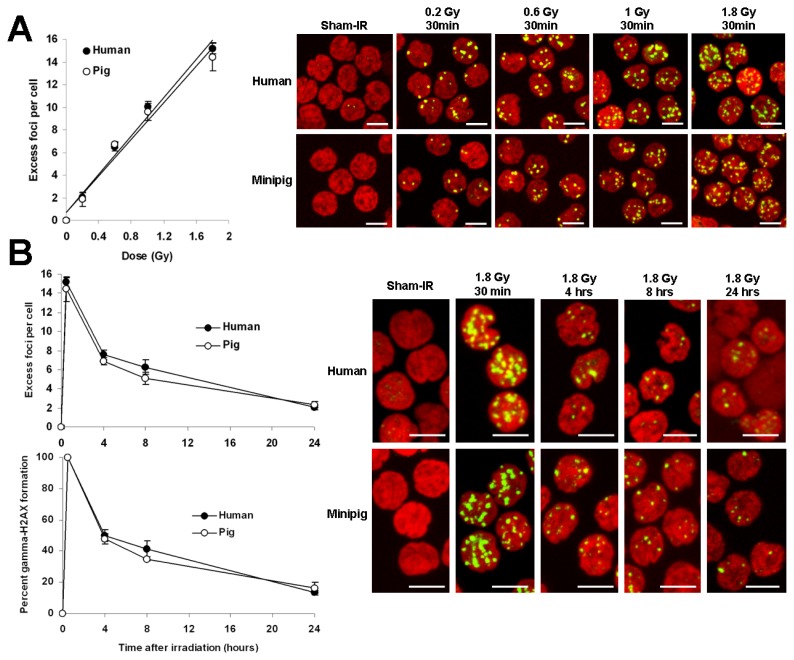
Comparison of γ-H2AX foci kinetics in human and minipig lymphocytes after *ex vivo* irradiation of blood samples. (**A**) Human and minipig blood was irradiated with 0.2, 0.6, 1 and 1.8 Gy and incubated at 37 °C for 30 min. Lymphocytes were purified, fixed, stained, and imaged. Left, data plotted as excess γ-H2AX fpc ± SD (*n =* 3) *vs.* dose. Right, representative images. Green, γ-H2AX; red, DNA stained with PI. Chi-square test for trend between human and minipig dose responses: χ^2^ = 0.0026, *p* = 0.9591 (no significant difference). *p* Values (Student’ *t*-test) between human and minipig responses for 0.2, 0.6, 1 and 1.8 Gy are 0.425, 0.541, 0.221 and 0.209 respectively (no significant difference); (**B**) Human and minipig blood samples were irradiated with 1.8 Gy and incubated at 37 °C for various lengths of time. Lymphocytes were prepared as described in panel A. Left, data plotted as excess γ-H2AX fpc ± SD (*n =* 3) (top chart) or as percentage of γ-H2AX foci remaining ± SD (*n =* 3) (bottom chart). Chi-square test for trend between human and minipig γ-H2AX kinetics: χ^2^ = 0.0013, *p* = 0.970 (no significant difference). *p* Values (Student’ *t*-test) between human and minipig responses for 0.5, 4, 8 and 24 h are 0.209, 0.042, 0.027 and 0.126 respectively (significantly different for 4 and 8 h). Right panel, Representative images of lymphocytes used for data acquisition. Green, γ-H2AX; red, DNA stained with PI. Excess γ-H2AX foci per cell were calculated by subtraction of the background (non-irradiated cells) and represent the amount of radiation-induced foci. Scale bars, 5 μm.

Preliminary to evaluating the swine model for γ-H2AX foci biodosimetry, we compared the foci incidence in both human and minipig lymphocytes 30 min to 24 h after *ex vivo* irradiation of blood samples with 0.2 to 1.8 Gy of cobalt-60 γ-radiation (Figure 1). The two species of lymphocytes were found to respond similarly to the irradiation doses tested, yielding linear relationships between the number of γ-H2AX foci and the irradiation dose of approximately 10 foci per cell (fpc) per Gy (10.00 ± 0.47 for human and 9.58 ± 0.74 for pig) at 30 min post-exposure (Figure 1A). γ-H2AX incidences between human and minipig were not significant (see Figure 1 legend for details). These results are comparable to the similar comparison made elsewhere between human and non-human primate (NHP) lymphocytes (Figure 1 of [13]).

The decrease in γ-H2AX foci incidence between 30 min and 24 h post-exposure was not statistically different between human and minipig lymphocytes (see Figure 1 legend for details) with both cell types exhibiting foci numbers at 24 h about 15% of those at 30 min (Figure 1B, 13.4% ± 1.4% in the human *vs.* 16.3% ± 0.3% in the minipig). At 24 h post exposure, the incidences of γ-H2AX foci in human (2.03 ± 0.21) and minipig (2.34 ± 0.31) lymphocytes were indistinguishable and about 10-fold above the control values (0.23 fpc in humans and 0.21 in minipigs). Again, these results were similar to those reported elsewhere for human and NHP blood samples [13,30–32]. Thus, the nearly identical responses observed between human and minipig lymphocytes suggests that the minipig may be a good model for developing a γ-H2AX-based radiation biodosimeter to study human radiation exposure.

### 2.2. Comparative Analysis of γ-H2AX Kinetics in Human and Minipig Fibroblasts

**Figure 2:**
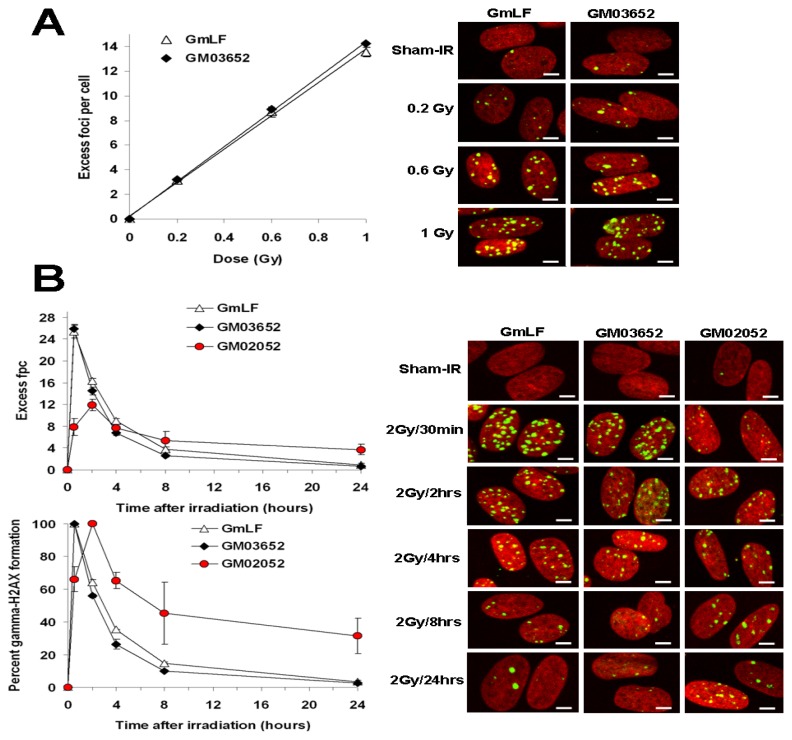
Comparison of γ-H2AX focus kinetics in human and minipig fibroblasts after *ex vivo* irradiation. (**A**) Cultures of human (GM03652) and minipig (GmLF) fibroblasts were irradiated with 0.2, 0.6, 1 and 1.8 Gy and incubated at 37 °C for 30 min. Cultures were fixed, stained, and imaged. Left, data plotted as excess γ-H2AX fpc ±SD (*n =* 3) *vs.* dose. Right, representative images. Green, γ-H2AX; red, DNA stained with PI. Chi-square test for trend between human and minipig dose responses: χ^2^ = 0.0018, *p* = 0.966 (no significant difference). *p* Values (Student’ *t*-test) between human and minipig responses for 0.2, 0.6 and 1 Gy are 0.647, 0.658 and 0.129 respectively (no significant difference); (**B**) Fibroblast cultures from normal (GM03652) or A-T diseased (GM02052) human subjects and from minipigs (GmLF) were irradiated with 2 Gy and incubated at 37 °C for various lengths of time, then prepared as described in panel A. Left panel, data plotted as excess γ-H2AX fpc ± SD (*n =* 3) (top chart) or as percentage of γ-H2AX foci remaining ±SD (*n =* 3) (bottom chart). Chi-square test for trend between human and minipig γ-H2AX kinetics: χ^2^= 0.3917, *p* = 0.5314 (no significant difference). *p* Values (Student’ *t*-test) between responses for 0.5, 2, 4, 8 and 24 h are 0.644, 0.085, 0.0517, 0.055 and 0.512 respectively (no significant difference). Right panel, Representative images of fibroblasts used for data acquisition. Green, γ-H2AX; red, DNA stained with PI. Scale bars, 10 μm.

To substantiate the similarity of relative sensitivities between the human and minipig, primary normal fibroblasts of human and porcine origin were compared for γ-H2AX kinetics and radiation sensitivity after *ex vivo* irradiation with Cesium 137 γ-rays (Figure 2A). Similar robust dose-dependent induction of γ-H2AX foci was found after γ-ray exposure of human and minipig fibroblasts. In addition, the kinetics of focus disappearance after 2 Gy was similar in human and minipig fibroblasts (Figure 2B). Even though γ-H2AX focus loss was slightly faster in human fibroblasts compared to the minipig fibroblasts at earlier times post-irradiation (55.88% ± 1.06% *vs.* 64.40% ± 1.53% foci per cell remaining at 2 h, 26.31% ± 2.97% *vs.* 35.35% ± 0.10% at 4 h, and 10 ± 0.16 *vs.*14.85% ± 0.83% at 8 h), the numbers of foci per human cell (0.86 ± 0.28) and per minipig cell (0.70 ± 0.06) were indistinguishable after 24 h (Figure 2B) when the levels had decreased to about 3% of the maximum (3.71% ± 0.94% *vs.* 2.72% ± 0.31% in minipig and human cells respectively) (Figure 2B). Interestingly, as previously observed with lymphocytes, the number of γ-H2AX foci was slightly higher with increasing radiation doses in human compared to porcine samples (Figures 1A and 2A); however statistical analysis shows that the difference was not significant (see Figure 2 legend for details). Such a discrepancy may be explained by the difference in DNA content [33,34]. Because the swine genome is estimated to be ~15% smaller than its human counterpart (2.7 Gigabases (Gbp) in pig *vs.* 3.2 Gbp in human [35–38]), the smaller relative target size in minipig cells would yield a correspondingly lower level of γ-H2AX foci, other factors being equal.

### 2.3. Comparative Analysis of Radiation Sensitivity in Human and Minipig Fibroblasts

**Figure 3:**
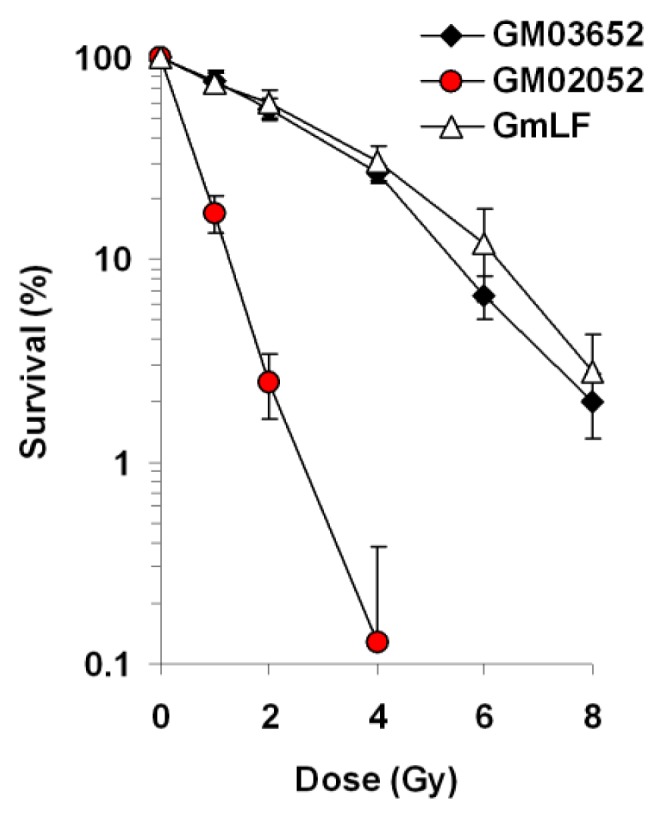
Clonogenic survival of human and minipig fibroblasts following exposure to ionizing radiation. Fibroblasts from normal (GM03652) or A-T diseased (GM02052) human subjects and from minipigs (GmLF) were plated onto 60 mm dishes, irradiated with 1, 2, 4, 6 and 8 Gy, those that survived to form colonies were counted after ~10 days. Percent survival ±SD (*n =* 3) was calculated relative to sham-irradiated cultures. Chi-square test for trend between human and minipig radiosensitivities: χ^2^ = 0.9830, *p* = 0.3215 (no significant difference). *p* Values (Student’ *t*-test) between human and minipig responses for 1, 2, 4, 6 and 8 Gy are 0.866, 0.563, 0.398, 0.185 and 0.436 respectively (no significant difference).

Because the fractions of remaining γ-H2AX foci were similar at 24 h post-exposure in both lymphocytes and fibroblasts of human and minipig, we hypothesized that both human and minipig cells may share a similar radiosensitivity. To examine this, we performed clonogenic survival assays on both swine (GmLF) and human (GM03652) primary fibroblast cultures, utilizing an ATM-deficient human cell line (GM02052) as a positive control for radiation sensitivity (Figure 3). ATM (ataxia telangiectasia mutated) protein is the central component of the signal transduction pathway for DSB repair [39] and those patients and their cells display DNA repair defects and high radiosensitivity [39].

The difference in radiosensitivity between human and minipig cells was not statistically different (see Figure 3 legend for details). The surviving fractions were 76.35% for human *vs.* 74.85% for minipig after 1 Gy; 55.71% for human *vs.* 59.61% for minipig after 2 Gy; 26.83% for human *vs.* 30.34% for minipig after 4 Gy; 6.65% for human *vs.* 11.87% for minipig after 6 Gy and 2% for human *vs.* 2.79% for minipig after 8 Gy. These values are similar to previously published data regarding survival of human and swine fibroblasts [28,40]. In contrast, ATM-deficient cells were hypersensitive to γ-rays (17.07%, 2.5% and 0.12% at 1, 2 and 4 Gy respectively), as previously reported [41]. Altogether, under our conditions the γ-H2AX kinetics *(i.e.*, DSB repair kinetics) and radiosensitivity were similar between human and swine fibroblasts.

Taken together, we found great similarity between human and minipig cells for the criteria we examined. These data are in agreement with findings that after cobalt 60 γ-irradiation, both the amount of DNA damage (measured by alkaline elution) and cell survival were similar between endothelial cells, smooth muscle cells and fibroblasts from human and swine origins [28].

These similar characteristics of human and pig cells in culture contrast with the sensitivities of the two organisms to exposure to ionizing radiation. While the LD_50_ of humans is estimated at 3.5–4 Gy, for minipigs it is considerably lower at 1.8 Gy. It appears then that the LD_50_s of the two species may not be due to inherent differences at the cellular level.

### 2.4. γ-H2AX Kinetics after Total-Body Irradiation of Minipigs

The nearly identical behavior between human and minipig cells supports the suitability of the minipig as a surrogate for human exposures and justifies the evaluation of *in vivo* irradiation of minipigs to evaluate the appropriateness of γ-H2AX as a radiation dosimeter. Twelve minipigs, divided into four groups, were subjected to a total body irradiation (TBI) protocol with zero (*n =* 4), 1.8 (*n =* 4), 3.8 (*n =* 2) and 5.0 (*n =* 2) Gy of cobalt-60 γ-rays. These doses were chosen to correspond to damage to the hematopoietic system (1.8 Gy, 3.8 Gy) with little or no damage to the GI tract and moderate damage to the GI tract (5.0 Gy) ([12] and Moroni, personal communication). Because blood draws are minimally invasive, they are still the method of choice for γ-H2AX detection in both human and animal models [13,29].

**Figure 4:**
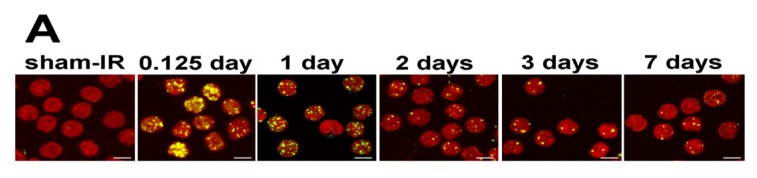

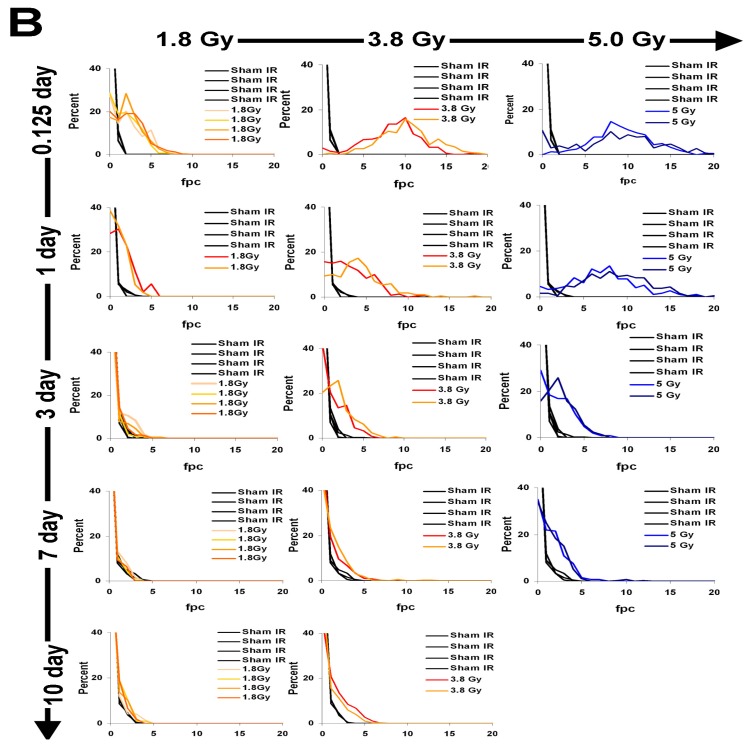
Distribution of γ-H2AX foci in peripheral blood lymphocyte populations taken at various times after exposure of minipigs to ionizing radiation. (**A**) Representative images of lymphocyte samples taken from minipigs 0.125, 1, 3, 7 and 10 days after 5 Gy TBI. Sham-irradiated animals were processed identically to the others. Green, γ-H2AX; red, DNA stained with PI; (**B**) The graphs show the percentage of lymphocyte populations with the noted number of γ-H2AX foci at the noted times and doses. Minipigs were exposed to 1.8, 3.8 and 5.0 Gy total body irradiation from a Co-60 source and allowed to recover. The graphs compare the samples taken from irradiated animals with unirradiated animals. The individual irradiated animals are noted by different color distribution curves (in each column). The focus distributions in the samples with the lowest and the highest foci per cell values are shown in red and blue respectively. Scale bars, 5 μm.

**Figure 5:**
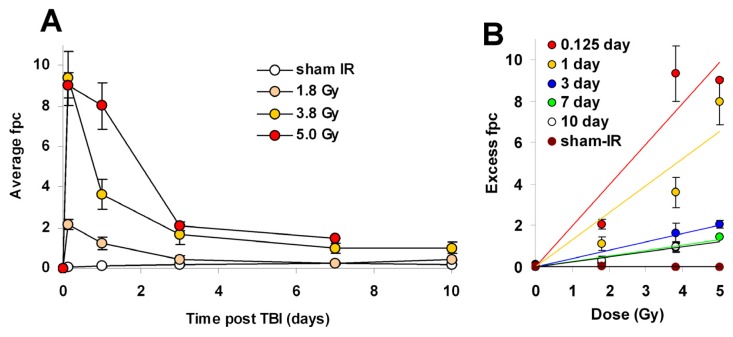
Kinetics for γ-H2AX focus formation and loss in peripheral blood lymphocytes following minipig total body irradiation (TBI). Foci were enumerated in images like those in Figure 4A for the above-noted times after zero, 1.8, 3.8, and 5 Gy. Values are expressed as average γ-H2AX foci per lymphocyte (average fpc) (**A**) or excess γ-H2AX foci per lymphocyte (excess fpc) ± SD (*n =* 4 for sham-IR and 1.8 Gy samples, *n =* 2 for 3.8 and 5.0 Gy samples) and plotted *vs.* time after irradiation (**A**); or dose (**B**). Animals irradiated with 5 Gy TBI died before the 10 day time point. Statistical differences (Student’s *t*-test) were observed between TBI and sham-IR for all times (*p* < 0.05) except for 7 days after 1.8 Gy (*p* = 0.632).

Lymphocytes in blood drawn at times ranging from 3 h to 10 days after TBI were purified and stained for γ-H2AX. The stained samples were imaged and γ-H2AX foci were enumerated in at least 200 lymphocytes from each sample (Figure 4A). The foci profiles of duplicate or quadruplicate minipig samples exhibited very similar distributions (Figure 4B). For minipigs exposed to 1.8 Gy, the focus profiles of the lymphocyte samples differed from the controls even 10 days post-exposure, however, the differences reached significance only until day 3 (Figure 5). For 3.8 and 5 Gy, values at 3 and 7 days post TBI were significantly above the controls. For 3.8 Gy TBI, significant differences from the controls were still present at 10 days post-TBI. The minipigs exposed to 5 Gy TBI were euthanized before 10 days to avoid pain, distress and suffering.

The kinetics of γ-H2AX focus formation and elimination for different TBI doses and recovery times were biphasic with a rapid decrease in focus numbers over the first 1–2 days (Figure 5). However, γ-H2AX focus numbers remained relatively stable after 3 days (Figure 5A). Fpc values for the 1.8-Gy samples differed significantly from those for the sham samples until 3 days post-exposure with 0.41 ± 0.18 fpc in the 1.8 Gy *vs.* 0.16 ± 0.06 in the sham. However, by 7 days post exposure, the fpc values of the 1.8 Gy and the sham samples were indistinguishable (0.26 ± 0.07 *vs.* 0.23 ± 0.06). In contrast, both the 3.8 and 5.0 Gy samples exhibited substantial responses for up to 10 and 7 days respectively (Figure 5A). The values at 7 days post-exposure were 1.48 ± 0.01 for 5.0 Gy and 0.98 ± 0.22 for 3.8 Gy *vs.* 0.23 ± 0.06 for sham-exposed, about 6.5 and 4.2 fold higher than the corresponding control values. At 10 days post-exposure the fpc value was 0.99 ± 0.27 for 3.8 Gy, about 5.5-fold above the corresponding control value (0.18 ± 0.00) (Figure 5A). However, while these animals responded similarly to the same doses of TBI, the SD values show that their responses were not identical (Figure 5A,B). A similar observation was previously made with the NHP model [13]. Interestingly, with 3.8 Gy-TBI, the fpc values remained similar between 7 and 10 days (0.98 ± 0.22 *vs.* 0.99 ± 0.27 at 7 and 10 days respectively). Similar observations were done in NHP lymphocytes after TBI [13]. The existence of these persistent foci suggests that a portion of irradiation-induced DSBs remained unrepaired in minipigs. While the molecular nature of these foci remains unknown, they may represent chromosome fragments or complex DNA lesions. Such lesions may be involved later on in cellular senescence and/or lead to chromosomal aberrations and micronuclei [42,43].

Elevated levels of γ-H2AX foci were found in NHP lymphocytes 14 days after 8.5 Gy, and in plucked hair bulbs 9 days after 8.5 Gy [13]. Also, elevated levels of γ-H2AX foci have been found in mouse skin 7 days after 1 Gy and in minipig skin 70 days after 50 Gy [19,44]. Residual foci were shown to be proportional to the initial irradiation doses, making it possible to utilize these values as a robust biodosimeter tool for analyzing TBI at times well after the initial exposure [13,44,45]. In human patients, the data for γ-H2AX levels have come from clinical studies evaluating the biological impact of clinical procedures utilizing ionizing radiation for diagnostics (X-ray examination, computed tomography, angioplasty) and cancer radiotherapy (see [29] for review). Studies with patients show a strong linear correlation between the mean number of γ-H2AX foci per lymphocyte and the integrated body radiation dose [46–51].

When regression curves for minipig TBI are calculated with the values of excess foci per cell (excess fpc) for sham-IR and TBI plotted *vs.* irradiation dose, the data for the different time points fall along straight lines (Figure 5B), particularly at 3 days and beyond. The curves for 3 h and 1 day are less straight probably because of differences in the time for transport of the samples. The absence of linear fit for the 0.125 and 1 day points may be due to the low number of animals combined with intra-animal and inter-animal variations and/or assay variations due to human error. Interindividual variability is a major issue regarding radiation biodosimetry studies, especially in humans where it has been extensively reported [46,52,53]. Therefore, variations in biological (age, intrinsic radiosensitivity) and environmental (smoking status in humans) factors, especially if they may affect DSB levels, should be taken into account. Automation and radiosensitivity assessment may help optimize biodosimetry assays [46,54,55]. Similar curves were developed with the NHP model [13]. As in NHPs, interpolated values for 1 Gy exposures are significantly different from sham-irradiated animals for up to 1 day post-exposure, after which no difference is detected between irradiated and sham controls ([13], Figure 5B). On the other hand, both NHPs and minipigs exposed to TBIs of 3.5 Gy and above exhibited values significantly greater than the sham-irradiated animals well beyond 1 day ([13] and Figure 5B). Thus, we conclude that γ-H2AX kinetics in lymphocytes following IR exposure is similar between the minipig and NHP models.

## 3. Experimental Section

### 3.1. Reagents

Fetal bovine serum (FBS) (Atlanta Biologicals, Norcross, GA, USA); Bovine serum albumin (BSA) (Sigma, St. Louis, MO, USA); Dulbecco’s modified eagle medium (DMEM) medium (Gibco, Grand Island, NY, USA); Anti γ-H2AX monoclonal antibody (Millipore, Billerica, MA, USA); Goat-anti mouse Alexa 488 (Invitrogen, Eugene, OR, USA); Penicillin/Streptomycin solution (Gibco, Grand Island, NY, USA); Trypsin (Gibco, Grand Island, NY, USA). Ficoll-Paque PLUS (GE Healthcare Bio-Sciences, Piscataway, NJ, USA); Vectashield (Vector Laboratories, Burlingame, CA, USA).

### 3.2. Cell Line and Cell Culture

Human skin fibroblasts cell lines GM03652 and GM02052 were obtained from ATCC (Manassas, VA, USA). Pig fibroblasts were derived from Gottingen minipig lungs cut in small pieces (1 mm or less) and trypsinized for 30 min at 37 °C before being transferred to a sterile Petri dish containing DMEM/15% FBS/Penicillin/Streptomycin. Fibroblasts were cultured in DMEM medium containing 15% fetal bovine serum, 100 units/mL Penicillin and 100 μg/mL Streptomycin in a humidified atmosphere with 5% CO_2_ at 37 °C.

### 3.3. Animal Housing and Care

Male Gottingen minipigs (4 months of age, 9–11 kg) were obtained from Marshall Bioresources (North Rose, NY, USA); the Gottingen minipig is the smallest minipig available specifically bred for biomedical purposes. Procedures were performed in accordance with protocols approved by the Armed Forces Radiobiology Research Institute (AFRRI) Institutional Animal Care and Use Committee; this institution is fully AAALAC-accredited. Minipigs were fed twice daily (Harlan Teklad Minipig diet 8753, Madison, WI, USA) according to individual weights and provider recommendations; water was provided ad libitum. Minipigs were singly housed in adjoining cages that allowed tactile, visual, olfactory and auditory contact through cage bars. Room temperature was kept between 64 and 79 °F (17.8 to 26.1 °C) and humidity between 30% and 70%. Environmental enrichment and stimulation were provided in the form of physical devices (treats, sanitized toys) and positive interactions with caretakers. To facilitate collection of blood samples, animals were quarantined for two weeks and implanted with a vascular access port (VAP) [56].

### 3.4. Animal Irradiation Procedures

After 3 weeks recovery from surgical implantation of the VAP, animals were subjected to total bilateral body irradiation using Cobalt-60, at a dose rate of 0.6 Gy/min. Animals were withheld food the night before irradiation and anesthetized with Telazol (tiletamine-zolazepam, 6 to 8 mg/kg IM) shortly prior to exposure. At the same time, animals were given a bolus injection of atropine (0.05 mg/kg IM) to decrease salivary secretions. Anesthetized animals were placed on a sling and continuously monitored throughout the irradiation period using closed circuit cameras. Calibration of the dose rate was done using Plexiglas cylinders of various diameters filled with water and each containing 12 alanine standard dosimeters. Signal was measured with an EPR spectrometer, calibrated with standard alanine dosimeters obtained from the National Institute of Standards Technology (NIST). Results were verified through an inter-comparison study with the UK National Physical Laboratory in Teddington. Doses actually given to the animals were also measured real time with an ionization chamber. No supportive care was given to the animals throughout the study [14].

### 3.5. Blood Sampling

Minipig blood samples were obtained from the VAP, from unanesthetized animals placed on a sling. Blood was collected with strictly aseptic technique in sample tubes containing EDTA and immediately stored on ice until further processing. Human blood samples were collected in heparin-coated tubes and obtained at the NIH blood bank from paid healthy volunteers who gave written informed consent to participate in an IRB-approved study for the collection of blood samples for *in vitro* research use. The protocol is designed to protect subjects from research risks as defined in 45CFR46 and to abide by all internal NIH guidelines for human subjects research (protocol number 99-CC-0168).

### 3.6. Sample Irradiation and Lymphocyte Isolation

Blood was aliquoted in 15 mL conical tubes and placed on ice until irradiated with a Cobalt-60 source (0.6 Gy/min). Irradiation was bilateral, at a dose rate of 0.6 Gy/min and at room temperature. After exposure, samples were incubated at 37 °C for various designated post-exposure times prior to PBMC isolation. Lymphocytes were isolated from whole blood samples by the commonly used Ficoll-Paque density gradient centrifugation. Lymphocyte separation was performed according to the manufacturer’s instructions (GE Healthcare Bio-Sciences, Piscataway, NJ, USA). Briefly, blood samples diluted 1:1 with PBS were layered onto equal volumes of Ficoll-Paque and centrifuged at 700× *g* for 25 min at room 20 °C. After centrifugation, the lymphocyte layers were washed once with cold PBS.

### 3.7. γ-H2AX Detection

Cells were prepared for immunocytochemistry as previously described [23]. Briefly, fibroblasts and purified lymphocytes were fixed for 20 min at room temperature (20–23 °C) with 2% paraformaldehyde then washed 3 times with PBS. Lymphocytes were spotted on slides by Cytospin after fixation. Both lymphocytes and fibroblasts were permeabilized with pre-chilled ethanol 70%, and stored at 4 °C overnight. Samples were then incubated in PBS for 15 min and blocked for 30 min with 5% BSA in PBS containing 0.5% Tween-20 and 0.1% Triton X-100 (PBS-TT). After 5 min in PBS, samples were incubated 2 h with the mouse monoclonal anti-γ-H2AX antibody (dilution 500 in 1% BSA in PBS-TT) followed by a 1 h incubation with a goat anti-mouse Alexa-488-conjugated IgG (dilution 500 in 1% BSA in PBS-TT). After one wash with PBS and 5 min incubation at 37 °C with RNase A 0.5 mg/mL in PBS, slides were mounted with mounting medium containing propidium iodide and sealed with nail polish.

Immunostaining was performed using the primary mouse monoclonal anti-γ-H2AX antibody (Millipore) and the secondary goat anti-mouse Alexa-488-conjugated IgG (Invitrogen, Eugene, OR, USA). Nuclei were stained with propidium iodide (PI). Laser scanning confocal microscopy was performed with a Nikon PCM 2000 (Nikon, Inc., Augusta, GA, USA). In order to count all γ-H2AX in nuclei on images, optical section (0.5 μm) through the thickness of the cells were imaged and combined in a maximum projection with Simple 32 software (Compix Inc., Cranberry, PA, USA). The foci were visually counted in at least 200 cells.

### 3.8. Clonogenic Survival Assay

Cell survival was assessed by colony formation assay. Cells were trypsinized and identical numbers of fibroblasts were plated on 60 mm dishes. Eight hours after seeding, cells were irradiated to 1–8 Gy in a Mark-1 γ-irradiator (JL Shepherd & Associates, San Fernando, CA, USA) at a dose rate of 2.2 Gy/min. After approximately 10 days of incubation, the colonies were fixed with methanol, stained with Coomassie and colonies with >20 cells were counted under a dissection microscope. Clonogenic survival curves were constructed from at least three independent experiments.

### 3.9. Statistical Analysis

All values in this study were expressed as mean ± 1 SD unless otherwise noted. The significant differences between the groups for TBI were analyzed by a Student’s *t* test and a *p* value of <0.05 was considered significant. The Chi-square test for trend was used to compare radiation responses in human and minipig cells with *p* < 0.05 being statistically significant.

## 4. Conclusions

The goal of this study was to validate the use of the minipig model for γ-H2AX-based biodosimetry following a wide range of acute doses of ionizing radiation. We used the γ-H2AX focus assay for comparative assessment of DNA damage formation and repair kinetics in human and minipig irradiated *ex vivo* and *in vivo*, as well as in a primary-cell culture system (*in vitro*). Our data show that kinetics of DSB repair in minipigs are similar to those in humans. The integrity of DNA repair mechanisms in this model is supported by the reported low rate in swine of cancer incidence [57], a pathology strictly associated with defective DNA repair [58]. The similarity of DSB repair kinetics (*i.e.*, γ-H2AX loss) between human and minipig was confirmed by the observation of comparable radiation sensitivity in primary cell line survival assays (*in vitro*). The minipig seems well suited as an alternative large animal model for γ-H2AX-based biodosimetry. The accentuated sensitivity to irradiation exhibited by the Gottingen minipig with respect to other large animal models (NHP, dogs), as demonstrated by a LD50/30 of 1.78 Gy, does not appear to be due to gross deficiency in DSB repair.
